# The association between dietary acrylamide intake and the risk of type 2 diabetes incidence in the Tehran lipid and glucose study

**DOI:** 10.1038/s41598-023-35493-x

**Published:** 2023-05-22

**Authors:** Firoozeh Hosseini-Esfahani, Niloofar Beheshti, Amene Nematollahi, Glareh Koochakpoor, Soheil verij-Kazemi, Parvin Mirmiran, Fereidoon Azizi

**Affiliations:** 1 Nutrition and Endocrine Research Center, Research Institute for Endocrine Sciences, Shahid Beheshti University of Medical Sciences, Tehran, Iran; 2 Department of Food Safety and Hygiene, School of Health, Fasa University of Medical Sciences, Fasa, Iran; 3 Maragheh University of Medical Sciences, Maragheh, Iran; 4 Endocrine Research Center, Research Institute for Endocrine Sciences, Shahid Beheshti University of Medical Sciences, Tehran, Iran

**Keywords:** Diseases, Endocrinology

## Abstract

This study aimed at investigating the association of acrylamide consumption with the incidence of type 2 diabetes (T2D) in adults. The 6022 subjects of the Tehran lipid and glucose study participants were selected. The acrylamide content of food items were summed and computed cumulatively across follow up surveys. Multivariable Cox proportional hazard regression analyses were performed to estimate the hazards ratio (HR) and 95% confidence interval (CI) of incident T2D. This study was done on men and women, respectively aged 41.5 ± 14.1 and 39.2 ± 13.0 years. The mean ± SD of dietary acrylamide intake was 57.0 ± 46.8 µg/day. Acrylamide intake was not associated with the incidence of T2D after adjusting for confounding variables. In women, a higher acrylamide intake was positively associated with T2D [HR (CI) for Q4: 1.13 (1.01–1.27), P trend: 0.03] after adjusting for confounding factors. Our results demonstrated that dietary intake of acrylamide was associated with an increased risk of T2D in women.

## Introduction

Acrylamide is produced during the preparation of high-carbohydrate foods such as potato chips, french-fries and breads which contain the amino acid asparagine^1^. When these foods are prepared at high temperatures (120 °C), sugar reacts with asparagine through Millard Reaction and acrylamide produced up to 1 mg/kg food^2^. Furthermore, acrylamide is also part of tobacco smoke^3^. Two main sources of public exposure to acrylamide are food and cigarette smoke.

Dietary acrylamide could be an important human health risk factor; previous studies observed neurotoxicity in workers exposed to acrylamide in the workplace^4,5^. Moreover, animal studies reported the relationship of acrylamide intake with significant carcinogenic and neurotoxic risk^6,7^. Epidemiological studies have issued conclusive evidence that dietary acrylamide exposure is associated with the risk of cancers in humans^8^; there are also studies that reported a significant association between dietary acrylamide intake and risk of renal, endometrial, and ovarian cancers^9^. International Agency for Research on Cancer considers acrylamide as a probable human carcinogen^10^. The effect of acrylamide in increasing oxidative DNA damage in target organs is one of the suggested mechanisms that explain the carcinogenicity of acrylamide^11^. Recent studies indicated that long-term high levels of dietary acrylamide intake may induce oxidative stress and chronic inflammation^12^. Prevalence of type 2 diabetes (T2D), a metabolic disorders characterized by increased blood glucose concentration, increase across the worldwide^13^. The etiology of diabetes remains mainly unspecified^14^; however, the common pathogenic mediators in the natural course of diabetes is oxidative stress^15^. These findings suggest this thesis that acrylamide intake may be actual in the onset of T2D. In the study by Wang et al., ingested acrylamide was associated with fasting plasma glucose elevation, oxidative DNA damage and lipid peroxidation in 3270 adults; the significant linear positive dose–response relationship was found between urinary acrylamide metabolite and fasting plasma glucose^16^. In the study by Lin et al., a 1 unit rise in Hb adducts of acrylamide was related to both reduced blood insulin and reduced insulin resistance; this association was stronger in subjects with a lower education level, smokers, whites and in subjects with body mass index (BMI) < 25 or > 30 kg/m^2^ than their counterparts^17^. The existence of such contradictory results led us to perform a cohort study aimed at investigating the association of acrylamide consumption with the incidence of T2D in a group of Iranian adults.

## Results

This study was done on 2707 men and 3315 women (n = 6022), respectively aged 41.5 ± 14.1 and 39.2 ± 13.0 years. The participants include 5563 censors and 549 incident cases of T2D during a median of 6.63 years of follow up. The mean ± SD of dietary acrylamide intake was 57.0 ± 46.8 µg/day (median: 46.4 µg/day).

The baseline characteristics of the subjects across quartiles of acrylamide intake are shown in Table 1. We found that those who consumed higher acrylamide intakes were younger (Q1: 43.2 ± 14.3, Q4: 37.9 ± 12.7, P < 0.001). The percentage of active smokers was higher in the upper quartiles of acrylamide intake (Q1-Q4: 13.0, 16.7, 22.2, 28.0%). The level of physical activity was higher in the upper quartiles of acrylamide intake (Q1: 495 ± 684, Q4: 640 ± 995, P < 0.001).

**Table 1:** Baseline characteristics of adult participants of the Tehran Lipid and Glucose Study across quartiles of acrylamide intake (n = 6022).

	Acrylamide intake				P
	Q1	Q2	Q3	Q4	
n	1504	1506	1506	1506	
Acrylamide (µg/day)	23.3 ± 5.98	38.9 ± 4.30	55.8 ± 6.03	106 ± 68.3	
Baseline age (years)	43.2 ± 14.3	40.8 ± 13.5	39.0 ± 13.1	37.9 ± 12.7	< 0.001
Women (%)	73.3	59.8	41.7	40	< 0.001
Smoking (%)					< 0.001
Non-smokers	69.6	66.5	58.7	51.8	
Passive smoker	17.4	16.3	19.1	20.1	
Active smoker	13.0	16.7	22.2	28.0	
Family history of diabetes (%)	14.4	12.3	14.4	14.3	0.60
Physical activity (MET/min/week)	495 ± 684	500 ± 733	587 ± 871	640 ± 995	< 0.001
Education (≥ 12 years)	20.2	25.5	29.9	32.2	< 0.001
BMI (kg/m^2^)	27.3 ± 4.92	27.0 ± 4.74	26.7 ± 4.70	26.9 ± 4.68	0.17
Waist circumference (cm)	88.2 ± 13.5	88.5 ± 13.0	88.8 ± 13.2	90.3 ± 12.8	0.007
Fasting blood glucose (mg/dl)	90.2 ± 9.39	90.4 ± 8.95	90.1 ± 8.91	90.7 ± 8.95	0.29
Systolic BP (mmHg)	111 ± 16.9	111 ± 16.7	112 ± 15.7	111 ± 14.8	0.23
Diastolic BP (mmHg)	73.9 ± 10.6	74.4 ± 10.9	74.63 ± 10.7	75.02 ± 10.5	0.10
Triglyceride (mg/dl)	134 ± 76.9	138 ± 79.1	138 ± 82.7	140 ± 88.0	0.27
HDL (mg/dl)	47.3 ± 11.4	45.7 ± 11.0	44.7 ± 10.7	44.7 ± 10.4	< 0.001
Energy intake (kcal/day)	1879 ± 481	2152 ± 465	2432 ± 495	2704 ± 610	< 0.001
Carbohydrate (% of energy)	58.4 ± 6.00	59.0 ± 5.37	59.1 ± 5.31	59.6 ± 5.66	0.001
Protein (% of energy)	14.6 ± 2.75	14.7 ± 2.49	14.7 ± 2.20	14.9 ± 3.24	0.37
Total fat (% of energy)	30.3 ± 5.66	29.7 ± 5.12	29.6 ± 5.09	29.3 ± 6.73	0.003
SFA (% of energy)	9.97 ± 2.45	9.62 ± 2.13	9.61 ± 3.40	9.45 ± 5.40	0.008
MUFA (% of energy)	10.2 ± 2.52	9.96 ± 2.05	9.96 ± 2.18	9.96 ± 5.25	0.14
PUFA (% of energy)	6.15 ± 1.89	6.02 ± 1.59	6.00 ± 1.54	6.05 ± 5.11	0.89
Fiber (g/1000 kcal)	10.9 ± 11.1	10.2 ± 4.01	9.75 ± 2.56	9.63 ± 2.78	< 0.001
Breads (g/day)	94.0 ± 42.0	142 ± 51.0	189 ± 73.0	256 ± 225	< 0.001
Confectionary products (g/day)	4.28 ± 3.82	6.28 ± 5.24	7.66 ± 7.73	9.19 ± 11.2	< 0.001
Roasted nuts (g/day)	4.03 ± 6.13	5.43 ± 8.12	6.92 ± 8.53	8.46 ± 13.4	< 0.001
Snacks (g/day)	5.25 ± 5.38	8.16 ± 7.66	11.6 ± 11.3	15.9 ± 28.1	< 0.001
Coffee (g/day)	1.56 ± 2.68	3.87 ± 5.43	7.87 ± 10.2	39.7 ± 65.7	< 0.001
Fast food (g/day)	9.01 ± 9.85	13.0 ± 12.6	15.7 ± 16.7	20.9 ± 28.9	< 0.001
Bakery products (g/day)	8.36 ± 8.99	13.0 ± 13.4	16.2 ± 17.1	19.3 ± 21.3	< 0.001

Values are mean ± SD unless otherwise listed. *Q* quartiles of choline intake, *BMI* body mass index, *BP* blood pressure, *HDL* high density lipoprotein cholesterol, *MUFA* mono-unsaturated fatty acids, *PUFA* poly-unsaturated fatty acids, *SFA* saturated fat.

Moreover, individuals in the upper quartiles of acrylamide intake had higher intake of total energy (Q1: 1879 ± 481, Q4: 2704 ± 610 kcal, P < 0.001), carbohydrate (Q1: 58.4 ± 6.00, Q4: 59.6 ± 5.66% of energy, P = 0.001) and lower total fat (Q1: 30.3 ± 5.66, Q4: 29.3 ± 6.73% of energy, P = 0.003) and saturated fatty acid intakes (Q1: 9.97 ± 2.45, Q4: 9.45 ± 5.40% of energy, P = 0.008). The distribution of FBG, SBP, DBP and TG did not differ across quartiles of acrylamide intake; however, the level of WC (Q1: 88.2 ± 13.5, Q4: 90.3 ± 12.8 cm, P = 0.007) was higher in the upper quartiles of acrylamide intake and the level of HDL-C (Q1: 47.3 ± 11.4, Q4: 44.7 ± 10.4 mg/dl, P < 0.001) was lower in higher quartiles of acrylamide intake.

**Figure 1:**
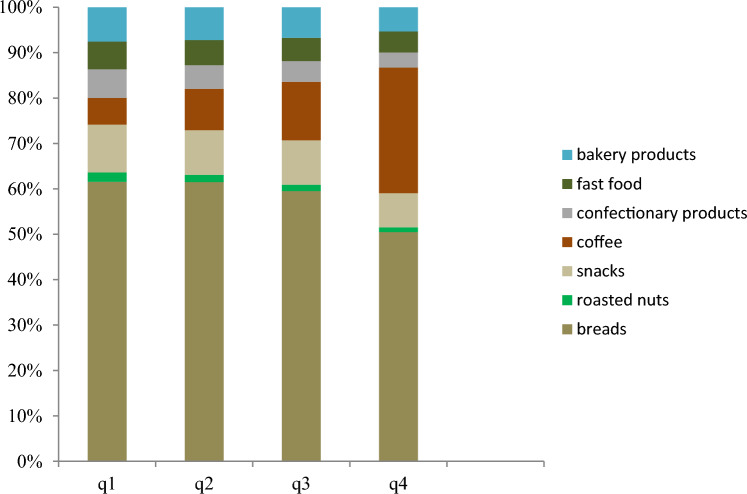
Contributors of dietary acrylamide intake from food items across quartiles of acrylamide intake.

The main contributors of acrylamide intake were for bread food items and coffee. The contribution of acrylamide intake from bread food items decreased from lower to upper quartiles of acrylamide intake, but the contribution of coffee increased from lower to upper quartiles of acrylamide intake (Fig. 1).

The Cox-proportional HRs for T2D based on quartiles of acrylamide intake is shown in Table 2. Acrylamide intake was positively associated with the incidence of T2D in model 1 (P for trend = 0.009); however, our results showed no association in model 2 after adjusting for confounding variables. Also the results of subgroup analysis were shown in this table. The association of acrylamide intake with T2D was not significant in subgroups of smoking categories. Our results indicated that after adjusting for confounding factors, a higher acrylamide intake was positively associated with T2D [HR (CI) for Q4: 1.13 (1.01–1.27), P for trend: 0.03] in women. In two subgroups of normal and overweight or obese individuals, there were no association between acrylamide intake and T2D.

**Table 2:** Hazard ratios (95% CI) for the association of acrylamide intake and type 2 diabetes incidence in all participants and subgroups.

	Total acrylamide intake				P for trend
	Q1	Q2	Q3	Q4	
Median intake (µg/day)	24.2	38.9	55.5	87.7	
All participants (n = 6022)					
Model 1	1	1.01 (0.94–1.08)	1.04 (0.97–1.12)	1.10 (1.02–1.18)	0.009
Model 2	1	1.00 (0.93–1.08)	1.02 (0.94–1.11)	1.06 (0.98–1.16)	0.13
Smoking*					
Non-exposed (n = 3714)	1	1.01 (0.92–1.11)	1.01 (0.92–1.11)	1.09 (0.98–1.22)	0.24
Passive smoker (n = 1204)	1	0.92 (0.77–1.10)	0.96 (0.80–1.16)	0.96 (0.79–1.16)	0.70
Active smoker (n = 1104)	1	1.08 (0.90–1.21)	1.15 (0.95–1.40)	1.14 (0.94–1.38)	0.18
Sex^†^					
Men (n = 2707)	1	0.93 (0.83–1.07)	0.96 (0.84–1.08)	0.96 (0.84–1.11)	0.85
Women (n = 3315)	1	1.02 (0.93–1.12)	1.05 (0.95–1.16)	1.13 (1.01–1.27)	0.03
BMI^††^					
< 25 kg/m^2^ (n = 2134)	1	1.05 (0.92–1.19)	1.04 (0.91–1.19)	1.07 (0.93–1.24)	0.37
≥ 25 kg/m^2^ (n = 3888)	1	0.97 (0.88–1.06)	1.00 (0.91–1.07)	1.05 (0.94–1.16)	0.27

Model 1: adjusted for age and sex, Model 2: Model 1 plus education, physical activity, family history of type 2 diabetes, body mass index, energy intake, triglyceride/high density lipoprotein cholesterol, smoking.

*Adjusted for age, sex, education, physical activity, history of type 2 diabetes, body mass index, energy intake, triglyceride/high density lipoprotein cholesterol.

^**†**^Adjusted for age, education, physical activity, family history of type 2 diabetes, body mass index, energy intake, Triglyceride/High density lipoprotein cholesterol.

^††^Adjusted for age, education, physical activity, family history of type 2 diabetes, energy intake, triglyceride/high density lipoprotein cholesterol.

## Discussion

The present study examined the association of acrylamide intake with T2D incidence in a group of adults in the TLGS after 6.63 years of follow up. Our findings showed that the contribution of acrylamide intake from bread food items was higher than other food groups including bakery products, coffee, roasted nuts, snacks, confectionary products and fast foods. Also acrylamide intake was positively associated with T2D incidence in total population; however, this association was not remained after adjusting for confounding variables. In this study we observed that a higher acrylamide intake was positively associated with the incidence of T2D in women, independent of other risk factors of T2D. There were no associations of acrylamide intake with T2D incidence in subgroups of smoking status, normal or overweight and obese individuals.

There is a large variation in estimation of dietary acrylamide intake in different countries. The median of dietary acrylamide intake ranged from 5.9 µg/day in 45–74 year old in Japan to 45 µg/day in a Swedish study among participants aged 45–73 years. A review study reported that European countries had higher dietary acrylamide estimation than other countries in the world especially in Asia, which might be due to differences in study designs, methods and dietary habits; however, the estimation of dietary acrylamide intake in our study was slightly higher than the Swedish participants, a European country. It might be due to eating foods in a way that results in higher acrylamide formation^18^.

In our study, the main food items responsible for acrylamide intake were respectively breads (52–62%), coffee (6–28%), snacks (7–10%), bakery products (5–7%), fast foods (4–6%) and roasted nuts (1–2%) in general population; while unlike our study, the expert committee on food additives reported that the principal food items accountable for acrylamide intake were respectively potato crisps (6–46%), potato chips (16–30%), coffee (13–39%), pastries and sweet biscuits (10–20%) and breads (10–30%)^19^. In our study, most participants consumed a high carbohydrate diet (~ 60% of energy intake) especially from refined sources (e.g. white bread)^20^. Also these results demonstrated that dietary acrylamide intake might be driven from staple foods such as breads that consumed in breakfast^18^. So food industry operators can apply methods to reduce acrylamide formation in bakery products such as using natural antioxidants obtained from spices and herb extracts in bakery products^21^.

Furthermore, it was demonstrated that the more important source of acrylamide exposure is smoking compared to dietary intakes^22^. Moreover acrylamide toxicity has a cumulative nature, so the chronic exposure at low doses via different routes is important. Also acrylamide has been proven to disrupt the endocrine system^23,24^, so low dose chronic of acrylamide exposure may create important health problems and it may be a contributing factor to induce diabetes. Acrylamide exposure at both high and low doses can change oxidative stress parameters and inflammation that can lead to intensive lipid peroxidation and atherosclerosis^25^.

To our knowledge, this is the first population-based study to report an association of dietary acrylamide intake and T2D. Unlike to our findings in all participants, previous epidemiological studies reported the relation between acrylamide exposure and glucose metabolism. Wang et al. found that urinary acrylamide metabolites were positively associated with FBG in a dose response manner in a general urban adult population. Also they indicated that acrylamide exposure is positively associated with FBG, oxidative DNA damage and lipid peroxidation^16^. In the American adult population, acrylamide exposure was associated with the insulin resistance and a decrease in insulin levels^17^. In the national health and nutrition examination survey (NHANES) the association of T2D and acrylamide biomarkers including hemoglobin adducts of acrylamide and glycidamide (HbAA and HbGA) was reported. HbAA was linearly and inversely associated with the risk of T2D, while HbGA/HbAA was positively and non-linearly associated with T2D prevalence^26^. HbAA and HbGA are considered as long-term exposure biomarkers of acrylamide in humans. Dietary acrylamide was positively correlated with HbAA and HbGA in previous studies; however, the results indicate that non-dietary exposure of acrylamide including tobacco smoke and environmental tobacco smoke (passive smoking) might increase the level of HbAA and HbGA which induce the significant result in this study^22^. People who smoke tobacco products have 3–4 times higher exposure levels of acrylamide than non-smoker people^22,27^.

There were increases in gluconeogenesis and glycogenolysis as well as reduction in glycolysis in the liver of rats. Change in gene expression may be responsible for these processes that may be associated with hypoinsulinemia and impaired insulin signaling pathway^28^. In addition acrylamide can increase adipocyte differentiation through upregulation of the expression of adiposity-related genes. In vitro and in vivo studies found that acrylamide exposure in daily life can induce weight gain and promote obesity. These outcomes of acrylamide can influence different metabolic disease like diabetes^29^.

In our study we found that a higher acrylamide intake increased the risk of T2D incidence in women by 13%, independent of other risk factors of T2D; this association was not observed in men.

The evidence on rats reported sex differences in FBG and insulin levels after AA exposure^28^. They reported increase in FBG, decrease in insulin level and pancreatic islet injury under 30 mg/kg body weight of AA exposure for three weeks in female rats, while similar results were observed after 50 mg/kg body weight AA exposure in male rats. These differences may be due to higher AA absorption (3.53 fold) in female than in male rats^28^.

Environmental chemicals could affect as antagonists or agonists through estrogenic or androgenic pathways. This pathway may disorder the number of hormone receptors and disrupt the levels of endogenous hormones. Dietary exposure of acrylamide was found to be associated with endocrine-related cancers in mammals. Also acrylamide intake might disorder thyroid homeostasis, altered sex hormone levels in cross-sectional studies^22,23^.

Our study has several strengths. We used large scale prospective association of acrylamide intake with T2D. Also we used a full range of high quality measurement of outcome biomarkers. The measurement of acrylamide intake was performed in frequent surveys and cumulatively summed, so changes in the exposure was considered in determining long time risk of T2D. The use of acrylamide intake is less expensive than acrylamide biomarkers to be applied as an exposure in large population.

However, some limitations should be regarded when interpreting these results. Although we had adjusted a range of potential confounders in our study, but some confounding factors including eating habits and food preparation methods were not estimated and they could not be adjusted in Cox regression models. Acrylamide exposure from cigarette smoking and occupation were not considered in this study since detailed occupational histories were not available in this study.

## Conclusion

The results of our study demonstrated that dietary intake of acrylamide was associated with an increased risk of T2D in women of the TLGS cohort. People especially women should be aware of the findings of our study considering the adverse effect of acrylamide exposure with T2D in their lives.

## Materials and methods

### Study population

**Figure 2:**
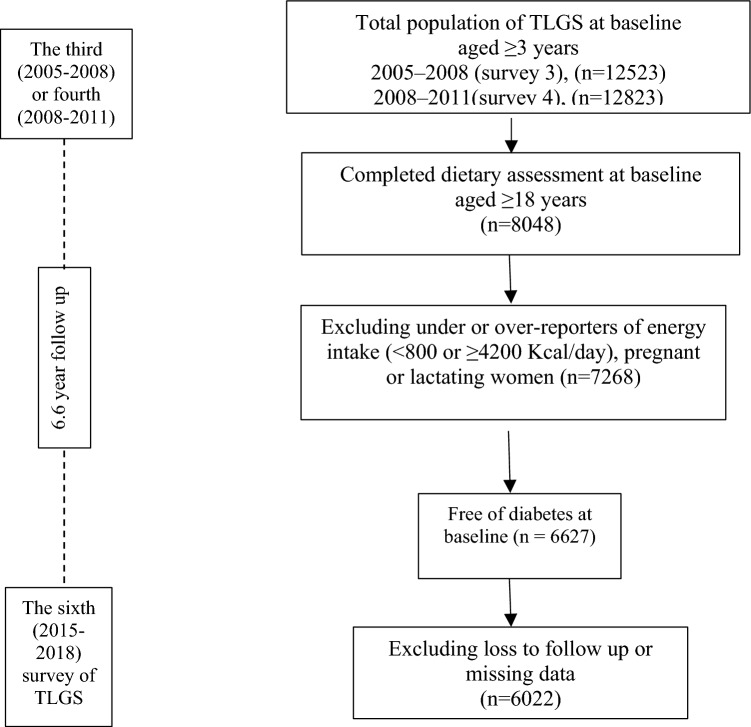
Outline of study participants’ selection.

Participants were selected from the Tehran lipid and glucose study (TLGS), an extensive population-based cohort study performed to find out risk factors for non-communicable diseases in a representative sample of Tehran, the capital of Iran^30,31^. The first survey was done from 1999 to 2001 on 15,005 individuals aged ≥ 3 years, using the composite stratified cluster random sampling technique. The follow-up surveys was done every 3 years; 2002–2005 (survey 2), 2005–2008 (survey 3), 2008–2011 (survey 4), and 2012–2015 (survey 5) and 2015–2018 (survey 6). Of subjects participating in surveys 3 and 4, we randomly selected 8048 individuals (≥ 18 years) with completed dietary assessment for this secondary analysis. We excluded subjects with under- or over-reporting of energy intake (< 800 or ≥ 4200 kcal/day)^32^ (n = 780), then a total of 7268 adult men and women with available anthropometric, dietary, and biochemical data were followed until survey 6 (participants entering at surveys 3 and 4 were respectively followed three and two times). Of these individuals, we precluded pregnant or lactating women and subjects diagnosed with T2D at baseline based on measurement of fasting blood glucose (FBG) or self-reported taking glucose-lowering drugs^33^ (n = 598). Finally, after precluding individuals missing any follow-up data (n = 498), 6022 participants remained and entered the analysis (Fig. 2).

All of the participants signed the written informed consent. The study was implemented in agreement with the Declaration of Helsinki rules and the study protocol was approved by the ethical committee of the Research Institute for Endocrine Sciences, Shahid Beheshti University of Medical Sciences, Tehran, Iran.

### Dietary assessment

Skilled nutritionists collected dietary data using a valid and reliable semi-quantitative food frequency questionnaire (FFQ). They questioned usual dietary intakes based on standard portion sizes during the last year through face-to-face personal interviews^34,35^. The consumption frequency of each food item was changed to daily intakes (g/day) using household measures. Due to the uncompleted Iranian food composition table (FCT), the United States Department of Agriculture (USDA) FCT was used to break down the nutrient composition of food items (e.g., bread, legume, nuts, white or red meat) not included in the Iranian FCT.

We used the values of acrylamide content reported in the analysis of 30 food items considering cooking methods and food preparation. The acrylamide content of traditional and industrial breads, bakery products (cakes and biscuits), confectionary products (pastries and chocolates), snacks (potato-based and corn-based), coffee powder, roasted nuts (almonds, pistachios, hazelnuts, peanuts and edible seeds), and fast foods (Pizza, hamburger and sausage) was evaluated. The acrylamide concentration of food was estimated using dispersive liquid–liquid micro-extraction system coupled with gas-chromatography mass spectrometry^36–38^. The nutrients and acrylamide content of food items were computed cumulatively across follow up surveys from the baseline survey until the point of the last follow-up visit or the time that diabetes diagnosed.

### Physical activity measurements

An interviewer questioned physical activity levels using Persian-translated modifiable activity questionnaire (MAQ). Previous study reported high reliability and moderate validity of this questionnaire^39^. Time and frequency of different intensity activities were collected as stated by routine activities of daily life over the past year. These data were changed into metabolic equivalent/minutes/week (MET/min/week)^40^.

### Blood pressure and anthropometric measurements

Skilled staff measured bodyweight using a digital scale (Seca 707) with a precision of 100 g. Subjects had minimum clothes and were barefoot. Height was measured by a tape measure with an accuracy of 0.5 cm in the standing position without shoes and straightened shoulders. Trained staff measured the waist circumference (WC) in the umbilical region after a normal exhalation without pressure on the body surface in the standing position and with the most miniature clothing to the nearest 0.1 cm.

Qualified physicians evaluated systolic and diastolic blood pressure (SBP, DBP) using a standard mercury sphygmomanometer. They asked participants to rest for 15 min, and then the physician measured blood pressure in the sitting position while setting the cuff on the right arm. They repeated this operation twice with an interval of 30 s. We took the average of two measures in the analysis.

### Biochemical analysis

Blood samples were collected between 7:00 to 9:00 a.m, after 12–14 h of overnight fasting. The samples were centrifuged 30 to 45 min after collection. The technician analyzed blood samples using Selectra 2 auto-analyzer at the TLGS research laboratory on the day of blood collection. They quantified FBG concentration using enzymatic colorimetric method and glucose oxidase technique (Vital Scientific, Spankeren, the Netherlands). In follow-up times, they perform the standard 2-h post-challenge blood glucose test using oral administration of 82.5 g glucose monohydrate solution (equivalent to 75 g anhydrous glucose) for all individuals who were not on glucose-lowering drugs.

High-density lipoprotein cholesterol (HDL-C) concentration was evaluated after precipitation of the apolipoprotein B-containing lipoproteins with phosphotungstic acid. Triglyceride (TG) level was assessed by enzymatic colorimetric tests using glycerol phosphate oxidase (Pars Azmoon Inc., Tehran, Iran). For glucose, inter-and intra-assay coefficients of variations were both 2.2%. For TG, inter-and intra-assay coefficients of variations were 1.6% and 0.6%, respectively^41^.

### Outcome definition

Incidence of T2D was determined as FBG concentrations ≥ 126 mg/dl or 2-h plasma glucose concentrations ≥ 200 mg/dl or self-reported taking glucose-lowering drugs (oral diabetes medication or insulin injections)^33^.

### Statistical analysis

We performed statistical analyses using the Statistical Package for Social Sciences (version 21.0; SPSS). A two-sided P value < 0.05 was considered statistically significant. We categorized data into quartiles of acrylamide intake. Chi-square test for categorical variables and one-way ANOVA for continuous variables were used to compare the mean and frequency of participants' baseline characteristics across quartiles of acrylamide intake. P for trend across acrylamide intake categories was performed by assigning continuous variables in a linear regression model. The normality of variables was checked by Kolmogorov–Smirnov test. Percentage of acrylamide from different food groups across quartiles of total acrylamide intake was computed. Multivariable Cox proportional hazard regression analyses were performed to estimate the hazards ratio (HR) and 95% confidence interval (CI) of incident T2D. The first quartile was assumed as the reference. The median of each quartile was utilized as a continuous variable to estimate the P-value of trends across quartiles of acrylamide intake in the Cox proportional hazard regression models. The confounders were selected based on literature; also, we apply each confounder in the univariable Cox regression model; a two-tailed P-value < 0.20 was practiced for specifying admission in the model.

Time to event was defined as the time between baseline and the event date (for event cases) or the last follow-up (for censored participants), whichever occurred first. The event date was defined as the mid-time between the follow-up visit date at which T2D was detected for the first time and the most recent follow-up visit before the diagnosis. Study participants were censored due to death, loss to follow-up, or non-occurrence of T2D before the end of follow-up. The Cox regression models were adjusted for several potential confounders. The analyses were performed with sex and age adjustment (model 1). Model 2 was adjusted for education levels (> 14 and ≤ 14 years), smoking (non-exposed, passive smoker, and active smoker), BMI (continuous), physical activity (continuous), family history of type 2 diabetes (yes, no), energy intake (continuous), TG/HDL ratio, plus model 1. Also multivariable Cox proportional hazard regression analyses were performed to estimate the hazards ratio (HR) and 95% confidence interval (CI) of incident T2D in subgroups of sex, smoking and BMI. The proportional hazards assumption was verified using the Schoenfeld residuals test and plot of log [− log (survival)] versus log (time) to see if they are parallel.

## Data Availability

The datasets generated during and/or analysed during the current study are available from the corresponding author on reasonable request.
